# Reviews

**Published:** 1875-01

**Authors:** 


### REVIEWS.

A Guide to the Practical Examination of Urine: For the use of Physicians and Students. By James Tyson, M.D., Hospital Lecturer on Pathological Anatomy in the University of Pennsylvania, etc., etc., with a Plate and numerous Illustrations. Philadelphia: Lindsay & Blakiston, 1875.

We have examined this work with as much attention as our time would permit, and think the author has covered the ground fully as expressed by him. He says, "While there were quite a number of comprehensive works of great value, and a smaller number of manuals or guides for the examinations of urine, the latter seemed altogether too limited, while the former are too bulky to be convenient for daily use."

Dr. Tyson—who, bye-the-bye, has been a valuable contributor to the pages of the Record—has enjoyed unusual facilities by several years' experience "in almost daily microscopical and chemical examinations of urine for others and himself, as well as teaching the subject in the University of Pennsylvania," giving him familiarity with the practical wants of physicians. It is well adapted to the wants of the busy practitioner, and will supply a place, in this respect, of positive utility.

Specific Diagnosis: A Study of Disease with Special Reference to the Administration of Remedies. By John M. Scudder, M.D., Professor of Pathology and the Practice of Medicine in the Eclectic Medical College, etc., etc. Cincinnati: Wilstach & Co., Printers, 1874.

This is the latest work of the author—fresh from the press—and is the twin brother of his "Specific Medication." We have had occasion to notice the peculiar views of Dr. Scudder as expressed in his writings, which we must truthfully say do not belong to the school of the Eclectics, so much as to the practical brain of Dr. Scudder himself. Dr. Scudder will find, perhaps, as many dissenters among his own school as any other in his new departure, and probably as many supporters in the latter as the former.

We believe his works abound with very valuable ideas, which will prove of service to the practitioner in his search for the truth. His work before us is an earnest and laudable effort to bring order out of confusion in the matter of diagnosis and therapeutics.

He concedes, as all physicians must, "that the study is difficult;" but contends that "it is difficult principally because it is new, and sufficient observations have not been made; that physicians have so long practiced by rote, and taken it for granted that there was no 'certainty in medicine,' that we have not the material we would wish for specific diagnosis."

There is always a charm about any system which guarantees "certainty"— which attempts to simplify the true, and which, at the same time, seeks to rectify and correct the errors and wrongs of other systems.

This is the object of the author; and whether he has only in part accomplished his purpose, or failed in toto, must be decided by the great tribunal of the medical world. He is at least entitled— as every earnest worker should be—to an impartial judgment, which, we trust, our readers will bestow. Whether they will accord to him more than earnestness in his efforts in the promulgation of his theories or not, we feel assured they will do themselves no harm by giving him a hearing.

THE PHYSICIANS' VISITING LIST FOR 1875. Twenty-fourth year of its Publication. Philadelphia: Lindsay & Blakiston. Sold by all Booksellers and Druggists.

This is one of the very best visiting lists published, and those who have not used it, do not know its great value. It is small, but convenient and ample for the purpose.

CASES OF HYSTERIA, NEURASTHENIA, SPINAL IRRITATION, AND ALLIED AFFECTIONS, WITH REMARKS. By George M. Beard, M.D. Chicago: J. J. Spalding & Co., Printers, 158 Clarke street. 1874.

THE TREATMENT OF MARASMUS, WHOOPING-COUGH, AND DEBILITY IN CHILDREN, BY ELECTRICITY. By George M. Beard, M.D., of New York City.

These pamphlets exhibit very clearly the value of electricity as a therapeutic agent in the treatment of nervous diseases. Dr. Beard has been a faithful and laborious investigator, and his pamphlets will repay the physician who desires to be informed on the subjects embraced in them.

THE LEGAL RELATIONS OF EMOTIONAL INSANITY. By E. Lloyd Howard, M.D., of Baltimore, Md. Philadelphia: Collins, Printer, 705 Jayne street. 1874.

The title will inform the reader of the design of the author, and may interest our readers.

THE HYPODERMIC USE OF QUININE: A Dangerous Experimental Medication, and rarely justifiable. By Stephen Rogers, M.D. (Reprint from the *New York Medical Journal*, September, 1874.) New York: D. Appleton & Co. 1874.

The author replies to an article of T. D. Leute, M.D., favoring the hypodermic use of quinine in intermittent fever, which he regards as dangerous. Dr. Rogers sustains his position with force, and we think with very great truth.

PROCEEDINGS OF THE MEDICAL ASSOCIATION OF THE STATE OF ARKANSAS: Fifth Annual Session. Held at Little Rock, October 20 and 21, 1874.

This is a small pamphlet, but nevertheless contains several well-written communications. We are indebted to Dr. Ed. Cross for the favor. W. T. G.

